# Novel insights on the role of VCAM-1 and ICAM-1: Potential biomarkers for cardiovascular diseases

**DOI:** 10.1016/j.amsu.2022.104802

**Published:** 2022-10-31

**Authors:** Rupinder Kaur, Varinder Singh, Pratima Kumari, Ravinder Singh, Hitesh Chopra, Talha Bin Emran

**Affiliations:** Chitkara College of Pharmacy, Chitkara University, Punjab, India; Department of Pharmacy, BGC Trust University Bangladesh, Chittagong, 4381, Bangladesh; Department of Pharmacy, Faculty of Allied Health Sciences, Daffodil International University, Dhaka, 1207, Bangladesh

**Keywords:** VCAM-1, ICAM-1, Proinflammatory cytokine, Antigen-presenting cells, Cell adhesion molecule

Cell adhesion molecules (CAM) including the intracellular adhesion molecule 1 (ICAM-1) and vascular cell adhesion molecule 1 (VCAM-1) are from the immunoglobulin family which regulates leukocyte adherence to endothelial muscles in acute or chronic inflammatory states [1]. TNF-α, a proinflammatory cytokine, triggers cell adhesion molecules, inflammatory molecules, and other cytokines [2]. The appearance level of these CAMs on the membrane of endothelial cells is a key factor in determining the comparable impact of VCAM-1 and ICAM-1 on the attraction of leukocytes in a particular disease state [1,2]. Leukocytes and endothelial cells are two cell types that express ICAM-1 an Ig-like cell adhesion molecule. Transcriptional regulation is primarily responsible for regulating ICAM-1 expression. The ICAM-1 is critically important in inflammation and the T-cell-mediated immune response. Antigen-presenting cells use it to stimulate T cells that recognize only MHC class II antigens, whereas other cells use it to stimulate cytotoxic T cells by associating with MHC class I. Leukocyte migration to the inflammatory site is done by ICAM-1 [3]. It has a significant role in inflammation-related processes and the defense system mediated by T-cells [1]. VCAM-1 is the other inflammatory cell adhesion molecule that may be therapeutically useful in immunological disorders and cancerous conditions. VCAM-1 controls the inflammatory vascular adhesion and leukocyte *trans*-endothelial migration [2].

The main cause of the majority of common cardiovascular problems is atherosclerosis, an inflammatory condition that develops gradually over time, it is characterized by plaque formation in the inner lining of the arteries, resulting in the hardening or narrowing of arteries. Increased inflammatory mediators and matrix metalloproteinases are secreted by migratory cells during this process, which causes plaque rupture and thrombus development [4]. Various studies have shown that measuring levels of ICAM-1, VCAM-1, and E-selectin can provide a good reflection of inflammatory processes occurring in the endothelium [5,6]. Although there are several ways to diagnose atherosclerosis, VCAM-1 represents a favorable target for molecular imaging-based non-invasive detection of atherosclerosis [7,8]. The initial stage of atherosclerosis is due to endothelial dysfunction, which is associated with ICAM-1 and VCAM-1 overexpression [6]. Upregulation of cellular adhesion molecules (ICAM-1 and VCAM-1), are among the phenotypic characteristics of endothelial dysfunction (VCAM-1). Indicative of the existence of atherosclerosis is an upregulation of the expression of intercellular monocyte adherence to the endothelium. Endothelial dysfunction, which has been associated with the development of atherosclerosis, has been found to be promoted by increased generation of reactive oxygen species (ROS) [9]. Endothelial cells produce chemokines and are specific for leucocytes. Before *trans*-endothelial migration, the attracted leucocytes perform the three procedures: Rolling adhesion, activation, and a strong adhesion-based arrest. After the leukocyte's release of cytokines like TNF alpha is slowed, leukocytes develop a loose bonding with selectins on the endothelial surface. This not only encourages the generation of additional cytokines, but it also leads to an overexpression of leukocyte adhesion molecules on the surface of endothelial cells [10]. It further leads to increased affinity of VCAM-1 to the integrins of leukocytes. E-selectin, VCAM-1, and ICAM-1 have a major role in the migration of lymphocytes, but still their prevalence in case of atherosclerotic lesions is not equal. Out of these three, VCAM-1 is an important cell adhesion molecule that has a significant part in both the beginning stages of atherosclerosis as well as its later stages. As a result, it is a straightforward target for the diagnosis of pathological situations [7,11].

Myocardial infarction is a condition in which the heart gets oxygen deprived, and blood flow to cardiac cells is reduced. Oxygen demand is higher than the oxygen supply. Patients with stagnant coronary flow had higher ICAM-1 and VCAM-1 level in the plasma [10]. Leukocyte chemotactic activity and recruitment are stimulated by the first inflammatory response in acute myocardial infarction (AMI), which results in neutrophil extravasation and infiltration, which is a major step of the inflammatory process in AMI [12]. IL-6, VCAM-1, and ICAM-1 serum levels were assessed in patients with myocardial infarction who were brought to the emergency. These three indicators of inflammation were demonstrated to be excellent indicators of heart failure following myocardial infarction. Specifically, they were considerably higher in patients with post-ST-segment elevation who experienced this condition [13]. In animal models of AMI, the neutrophil buildup is believed to lead to the no-reflow phenomena by inducing microvascular plugging that ultimately leads to further microvascular dysfunction and activation and release of mediators that induce vasoconstriction [12]. It is easy to correlate the expressions of cell adhesion molecules in the blood with their levels on the cell surface because these molecules have the probability of detaching themselves from the cell surface and further enter to the bloodstream without any intermediate steps [14]. These serum markers contributions to the acute coronary syndrome's inflammatory process, as well as any potential immunological pathways that may be linked to this condition, have been to be identified, as have their likely roles in the diagnosis and prognosis of this condition in its various clinical manifestations [12,14]. Acute myocardial infarction and other coronary syndromes have been linked to ICAM-1 and VCAM-1 in several investigations. Lino et al. found that individuals with heart failure had significantly elevated levels of the adhesion molecules ICAM-1 and VCAM-1 [15]. Therefore, ICAM-1 and VCAM-1 are related with the progression of myocardial infarction.

Furthermore, in the case of hypertension, endothelial dysfunction is an important abnormality observed. High blood pressure along with inducing endothelium damage, also causes vascular and systemic subclinical inflammation and is the major cause of cardiovascular complications and disease [16]. Cell infiltration into the vessel wall is caused by inflammatory stimulation during endothelial dysfunction, and VCAM-1 is crucial to this process. Numerous investigations employing hypertensive animal models, notably which are produced by Angiotensin II (Ang II), have found that the levels of VCAM-1, in protein and mRNA are elevated in the vascular system namely the aorta and mesenteric artery [13]. Serum ICAM-1, and VCAM-1 play a function in vasculature-related inflammation and activation of endothelial cells, but blood pressure fluctuation is a discrete risk factor of the cardiovascular system equally as foremost as in the management of BP [17]. A study by Lang et al., shows that ICAM-1 is essential for vascular dysfunction and hypertension induced by Angiotensin II. Hypertension is caused by obesity-associated adipocyte dysfunction, which is linked to the fact that adipokines have endocrine and paracrine repercussions on endothelium, smooth muscle cells of the cardiovascular system, along with macrophages. Moreover, researchers found evidence that ICAM-1 levels in the mRNA along with protein were remarkably elevated in aortas that had been infused with Ang II as compared to the samples that served as controls. Another study that was carried out by Kuroda and colleagues reveals that there is a notably positive association between the soluble VCAM-1 level and LV mass indices, as well as hypertension. This association was found to be significant. Those who suffer from left ventricle hypertrophy have higher levels of circulating soluble VCAM-1 than those who do not suffer from left ventricle hypertrophy [18]. Compared to normotensive adults, hypertensive patients have been found to have increased amounts of the soluble VCAM-1 and ICAM-1 in their blood [19]. The role that ICAM-1 and VCAM-1 play in the development of cardiovascular disorders is demonstrated in Fig. 1. It is of utmost importance to first identify the cardiovascular risk to prevent cardiovascular complications including hypertension, atherosclerosis, myocardial infarction, stroke, etc.

**Fig. 1:**
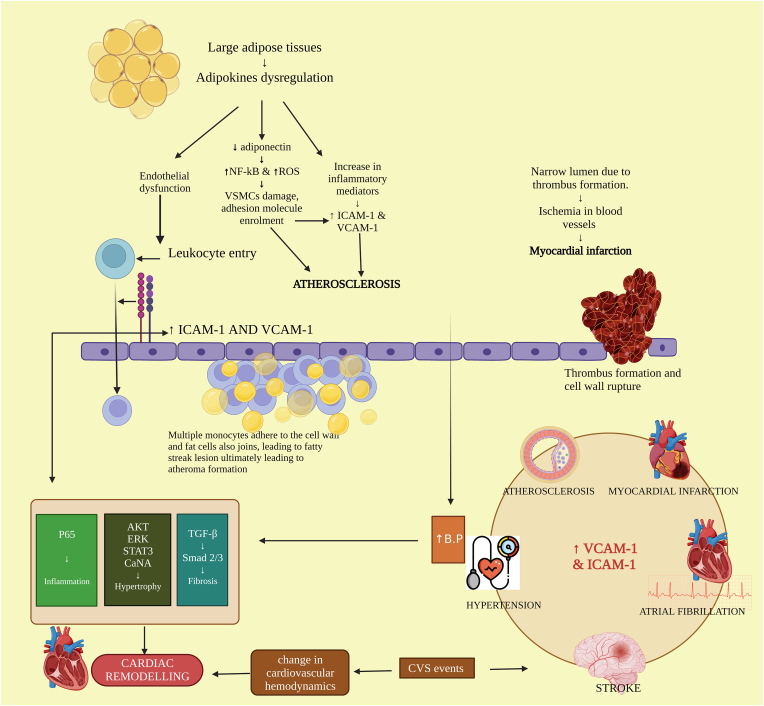
Schematic representation of the involvement of ICAM and VCAM in various cardiovascular diseases. So far, better knowledge and a picture of the pathological roles of ICAM-1 and VCAM-1 in humans will drive us to conduct more clinical studies to validate their threatening effect on vascular function. Furthermore, accessibility to new biomarkers/targets (ICAM-1 and VCAM-1) could provide considerable improvements in patient risk assessment and may further act as a therapeutic molecule in the management and preventive strategies of various cardiovascular diseases.

## Provenance and peer review

Not commissioned, internally peer-reviewed.

## Data statement

No specific data collected for the above manuscript.

## Ethical approval

Not applicable.

## Sources of funding

This study received no specific grant from any funding agency in the public, commercial, or not-for-profit sectors.

## Author contribution

Rupinder Kaur: Conceptualization, Data curation, Writing- Original draft preparation, Writing- Reviewing and Editing. Varinder Singh: Data curation, Writing- Original draft preparation, Writing- Reviewing and Editing. Pratima Kumari: Data curation, Writing- Original draft preparation, Writing- Reviewing and Editing. Ravinder Singh: Writing-Reviewing and Editing, Visualization, Supervision. Hitesh Chopra: Writing- Reviewing and Editing, Visualization, Supervision. Talha Bin Emran: Writing- Reviewing and Editing, Visualization, Supervision.

## Registration of research studies

1. Name of the registry: Not applicable.
2. Unique Identifying number or registration ID: Not applicable.
3. Hyperlink to your specific registration (must be publicly accessible and will be checked): Not applicable.

## Guarantor

Talha Bin Emran, Ph.D., Associate Professor, Department of Pharmacy, BGC Trust University Bangladesh, Chittagong 4381, Bangladesh. T: +88-030-3356193, Fax: +88-031-2550224, Cell: +88-01819-942214. https://orcid.org/0000-0003-3188-2272. E-mail: talhabmb@bgctub.ac.bd.

## Consent

Not applicable.

## Declaration of competing interest

All authors report no conflicts of interest relevant to this article.

## References

[1] Sans M., Panés J., Ardite E., Elizalde J.I., Arce Y., Elena M., Palacín A., Fernández–Checa J.C., Anderson D.C., Lobb R., Piqué J.M. (1999). VCAM-1 and ICAM-1 mediate leukocyte-endothelial cell adhesion in rat experimental colitis. Gastroenterology.

[2] Kong D.H., Kim Y.K., Kim M.R., Jang J.H., Lee S. (2018). Emerging roles of vascular cell adhesion molecule-1 (VCAM-1) in immunological disorders and cancer. Int. J. Mol. Sci..

[3] Van De Stolpe A., Van Der Saag P.T. (1996). Intercellular adhesion molecule-1. J. Mol. Med..

[4] Santos J.C., Cruz M.S., Bortolin R.H., Oliveira K.M., Araújo J.N., Duarte V.H., Silva A.M., Santos I.C., Dantas J.M., Paiva M.S., Rezende A.A. (2018). Relationship between circulating VCAM-1, ICAM-1, E-selectin and MMP9 and the extent of coronary lesions. Clinics.

[5] Goel S., Miller A., Agarwal C., Zakin E., Acholonu M., Gidwani U. (2015).

[6] Galkina E., Ley K. (2007). Vascular adhesion molecules in atherosclerosis. Thromb. Vasc. Biol..

[7] Cybulsky M.I., Iiyama K., Li H., Zhu S., Chen M., Iiyama M., Davis V., Gutierrez-Ramos J.C., Connelly P.W., Milstone D.S. (2001). A major role for VCAM-1, but not ICAM-1, in early atherosclerosis. J. Clin. Investig..

[8] Thayse K., Kindt N., Laurent S., Carlier S. (2020). VCAM-1 target in non-invasive imaging for the detection of atherosclerotic plaques. Biology.

[9] Habas K., Alterations Shang L. (2018). Intercellular adhesion molecule 1 (ICAM-1) and vascular cell adhesion molecule 1 (VCAM-1) in human endothelial cells. Tissue Cell.

[10] Turhan H., Saydam G.S., Erbay A.R., Ayaz S., Yasar A.S., Aksoy Y., Basar N., Yetkin E. (2006). Increased plasma soluble adhesion molecules; ICAM-1, VCAM-1, and E-selectin levels in patients with slow coronary flow. Int. J. Cardiol..

[11] Cai J., Zhang M., Liu Y., Li H., Shang L., Xu T., Chen Z., Wang F., Qiao T., Li K. (2020). Iron accumulation in macrophages promotes the formation of foam cells and development of atherosclerosis. Cell Biosci..

[12] Benson V., McMahon Clare, Claude A., Lowe H. (2007). ICAM-1 in acute myocardial infarction: a potential therapeutic target. Curr. Mol. Med..

[13] Troncoso M.F., Ortiz-Quintero J., Garrido-Moreno V., Sanhueza-Olivares F., Guerrero-Moncayo A., Chiong M., Castro P.F., Garcia L., Gabrielli L., Corbalan R., Garrido-Olivares L. (2021). VCAM-1 as a predictor biomarker in cardiovascular disease. Biochim. Biophys. Acta, Mol. Basis Dis..

[14] Macías C., Villaescusa R., delValle L., Boffil V., Cordero G., Hernández A., Hernández P., Ballester J.M. (2003). Endothelial adhesion molecules ICAM-1, VCAM-1 and E-selectin in patients with acute coronary syndrome. Rev. Esp. Cardiol..

[15] Lino D.O., Freitas I.A., Meneses G.C., Martins A.M., Daher E.F., Rocha J.H., Silva G.B. (2019). Interleukin-6 and adhesion molecules VCAM-1 and ICAM-1 as biomarkers of post-acute myocardial infarction heart failure. Braz. J. Med. Biol..

[16] Ciobanu D.M., Mircea P.A., Bala C., Rusu A., Ş Vesa, Roman G. (2019). Intercellular adhesion molecule-1 (ICAM-1) associates with 24-hour ambulatory blood pressure variability in type 2 diabetes and controls. Cytokine.

[17] Stevens S.L., Wood S., Koshiaris C., Law K., Glasziou P., Stevens R.J., McManus R.J. (2016). Blood pressure variability and cardiovascular disease: systematic review and meta-analysis. BMJ.

[18] Lang P.P., Bai J., Zhang Y.L., Yang X.L., Xia Y.L., Lin Q.Y., Li H.H. (2020). Blockade of intercellular adhesion molecule-1 prevents angiotensin II-induced hypertension and vascular dysfunction. Lab. Invest..

[19] Kuroda Y.T., Komamura K., Tatsumi R., Mori K., Yoneda K., Katayama Y., Shigemoto S., Miyatake K., Hanafusa T. (2001). Vascular cell adhesion molecule-1 as a biochemical marker of left ventricular mass in the patients with hypertension. Am. J. Hypertens..

